# A novel RNAseq–assisted method for MHC class I genotyping in a non-model species applied to a lethal vaccination-induced alloimmune disease

**DOI:** 10.1186/s12864-016-2688-0

**Published:** 2016-05-17

**Authors:** Wiebke Demasius, Rosemarie Weikard, Frieder Hadlich, Johannes Buitkamp, Christa Kühn

**Affiliations:** Institute for Genome Biology, Leibniz Institute for Farm Animal Biology (FBN), 18196 Dummerstorf, Germany; Institute of Animal Breeding, Bavarian State Research Center for Agriculture, 85586 Grub, Germany; Faculty of Agricultural and Environmental Sciences, University Rostock, 18059 Rostock, Germany

**Keywords:** MHC class I, MHC typing, Expression levels, Cattle, RNAseq, Bovine neonatal pancytopenia (BNP)

## Abstract

**Background:**

MHC class I genotyping is essential for a wide range of biomedical, immunological and biodiversity applications. Whereas in human a comprehensive MHC class I allele catalogue is available, respective data in non-model species is scarce in spite of decades of research.

**Results:**

Taking advantage of the new high-throughput RNA sequencing technology (RNAseq), we developed a novel RNAseq-assisted method (RAMHCIT) for MHC class I typing at nucleotide level. RAMHCIT is performed on white blood cells, which highly express MHC class I molecules enabling reliable discovery of new alleles and discrimination of closely related alleles due to the high coverage of alleles with reads. RAMHCIT is more comprehensive than previous methods, because no targeted PCR pre-amplification of MHC loci is necessary, which avoids preselection of alleles as usually encountered, when amplification with MHC class I primers is performed prior to sequencing. In addition to allele identification, RAMHCIT also enables quantification of MHC class I expression at allele level, which was remarkably consistent across individuals.

**Conclusions:**

Successful application of RAMHCIT is demonstrated on a data set from cattle with different phenotype regarding a lethal, vaccination-induced alloimmune disease (bovine neonatal pancytopenia), for which MHC class I alleles had been postulated as causal agents.

**Electronic supplementary material:**

The online version of this article (doi:10.1186/s12864-016-2688-0) contains supplementary material, which is available to authorized users.

## Background

The major histocompatibility complex (MHC) plays a fundamental role in immune response [1, 2]. The MHC comprises three classes: class I, class II and class III [3]. The main role of MHC molecules is the presentation of antigens, i.e., short peptide fragments derived from pathogens to the appropriate T cell receptor. MHC class I molecules preferentially display pathogens from cytosolic origin, e.g., viral peptides, and are ligands for antigen receptors of cytotoxic T cells. A comprehensive summary can be found in [4]. Within MHC class I, classical and non-classical genes can be distinguished. A high degree of diversity at the MHC is pivotal for recognition of the plethora of potential antigens. To cope with the high number of different antigens two mechanisms of diversification at individual and population level had evolved: first the MHC is polygenic and second it is highly polymorphic, i.e., often different numbers of specific MHC genes per haplotype occur and some are among the most polymorphic genes known [3]. Humans have an invariable number of three highly polymorphic, co-dominantly expressed classical MHC class I genes [5]. In contrast, in cattle, a divergent number of genes per MHC class I haplotype occurs [6]. In addition, in cattle there is no clear distinction between MHC class I genes due to high sequence similarity between alleles assigned to different genes [6]. These features together with the high degree of polymorphism substantially impeded MHC class I allele recognition and MHC class I genotyping using gene specific primers or genomic sequencing. Together with restricted resources compared to human, this resulted in the limited number of e.g., 97 MHC class I alleles for *Bos taurus* deposited in the Immuno Polymorphism Database (IPD-MHC, www.ebi.ac.uk/ipd/mhc/bola, [7]) compared to 3192 alleles for human HLA-A, 3977 alleles for HLA-B and 2740 alleles for HLA-C genes ([8] ftp://ftp.ebi.ac.uk/pub/databases/imgt/mhc, accession 2015/09/11). The previous obstacles in MHC genotyping might be overcome by new experimental techniques of deep RNA sequencing that enable the development of novel, comprehensive methods for allele discovery and diagnostics at the MHC locus. This is of particular interest in species with complex MHC class I haplotypes and/or a limited allele catalogue and unknown haplotype configuration.

Historically, MHC genotyping has been performed by serological, cellular or molecular methods. These are increasingly replaced by sequence-based analyses, mostly relying on DNA or RNA based diagnostics [9]. These techniques are easier to standardize and do not require the laborious antisera production and exchange between laboratories. Initially methods directed at detecting specific groups of MHC alleles using targeted primers for DNA or cDNA amplification and subsequent Sanger sequencing were in use. The problems and limitations with these methods are: i) only known single loci can be monitored, ii) a high degree of polymorphism disables unequivocal allele identification, if the individual is heterozygous at more than one position in the targeted gene, iii) specific tests for each gene or even allele group have to be developed. Increasingly, next-generation sequencing technology with mass sequencing of PCR amplification products is adapted to overcome some of those problems with this MHC genotyping strategy e.g., [10], although many new typing methods still carry the limitations associated with PCR amplification of specific target regions [10]. However, deep sequencing methods of whole genomes/transcriptomes now provide raw data for a, comprehensive survey of all (expressed) MHC alleles of an individual. Initially, two methods have been described for Human Leukocyte Antigen (HLA) typing using short sequence reads acquired by deep transcriptome sequencing (RNAseq) [11, 12]. This concept has been further extended to the use of whole genome sequencing data and exome sequencing [9, 13, 14]. However, these MHC typing methods build upon the available comprehensive collection of MHC alleles in human, which can be assumed to cover almost all alleles present in the population. This assumption of an almost complete catalogue of MHC alleles across breeds/populations is not valid in cattle or other non-model species. Thus, we further extended the initial whole genome/transcriptome-based approach by developing a novel MHC class I typing method, which also enabled a description of new alleles. This is essential for a fully comprehensive RNAseq based MHC class I typing in species with no or limited information on MHC class I alleles in the population. We applied the novel RNAseq-assisted method (RAMHCIT) in the investigation of the causal background of Bovine neonatal pancytopenia (BNP) for MHC class I typing of BNP- inducing and non-BNP control dams and the MDBK cell line.

BNP is a newly discovered, fatal, alloimmune/alloantibody-mediated disease of neonatal calves [15]. BNP is induced by ingestion of colostrum from cows vaccinated with a specific inactivated vaccine (PregSure®BVD, Pfizer Animal Health) against Bovine Virus Diarrhea (BVD) [16–18], which includes a novel, very potent nanoparticle based adjuvant. Alloantibodies, presumably induced after vaccination with PregSure®BVD, bind to MHC class I cell surface proteins of calf’s leukocytes and also to the Madin-Darby bovine kidney (MDBK) cell line [19, 20], which was used for virus culture during PregSure®BVD production. This suggested that contaminating MHC class I antigens in the vaccine might elicit pathogenic alloantibodies in some cows supporting the genetic predisposition documented for BNP [21–23]. However, a number of controversial observations contradict the hypothesis of single specific MDBK MHC class I alleles being monocausal for BNP. For example, given the high level of polymorphism at the MHC class I locus, a high proportion of individuals should lack a common allele with the MDBK cell line and according to the hypothesis should produce BNP colostrum. This is in contrast to the rather limited incidence of BNP cows given the large number of vaccinated individuals [24]. In addition, no single causal MHC class I alleles have been identified up to now.

Recent studies suggested that cross-reactivity of MHC class I allele specific antibodies and quantity of anti-MHC class I antibodies might be background for the variation in MHC class I mediated BNP responsiveness [25]. However, the studies relied upon the catalogue of existing MHC class I alleles and PCR amplification of MHC class I sequences. Given the low number of MHC class I alleles reported for cattle compared e.g., to human, it has to be expected that in cattle a very substantial number of alleles is still unreported. Thus it could not be excluded that a pivotal MHC class I allele crucial for BNP is not yet detected due to technical limitations.

These and other observations prove that the exact pathogenesis of the vaccine-induced BNP is not yet elucidated. New methods exploiting whole transcriptome sequencing data might be one step for improving the knowledge on BNP aetiopathology by providing a comprehensive description of all expressed MHC class I alleles of BNP-inducing and non-BNP-inducing dams.

Our novel RNAseq-assisted method (RAMHCIT) for MHC class I enabled typing of BNP- inducing and non-BNP control dams and the MDBK cell line. Tests for Mendelian inheritance of alleles and haplotypes within half sib and full sib families provided evidence that the method is capable to correctly identify published classical and non-classical MHC class I alleles. The method also discovered novel classical and non-classical MHC class I alleles, which demonstrates its capacity to add new sequence information to the currently available Bovine Leukocyte Antigen (BoLA) sequence catalogue (IPD-MHC database). Regarding aetiopathology of BNP, the data obtained by applying the new method RAMHCIT indicate that additional factors other than structural differences in MHC class I alleles are involved in BNP aetiopathogenesis.

## Methods

### Samples

All experimental procedures were carried out according to the German animal care guidelines and were approved and supervised by the relevant authorities of the State Mecklenburg-Vorpommern, Germany (State Office for Agriculture, Food Safety and Fishery Mecklenburg-Western Pommerania (LALLF M-V), 7221.3-2.1-005/11). For the study, six lactating and six non-lactating cows (Additional file 1), three to five years old, were investigated. Except one Holstein cow, all individuals were F_2_ cows from a German Holstein x Charolais crossbred population [26]. For this population, evidence had been provided for a genetic predisposition for clinical and subclinical BNP [21, 22]. Three different groups of cows were differentiated according to BNP incidence in their offspring. Group BNP-C (*n* = 4) comprised cows which had calves with clinical BNP and group BNP-H (*n* = 5) contained cows which had calves showing no clinical BNP, but hematological deviations from the average of the peer group. Finally, our data also included three control cows from sire lines unaffected by BNP and with calves lacking any clinical or hematological indications on BNP. Eight F_2_ cows were F_2_ full sibship and the three further F_2_ individuals were half sibs, which enabled MHC class I haplotype tracking within families. All 12 cows had received a basic vaccination with an inactivated BVD vaccine PregSure®BVD (Pfizer Animal Health) according to the manufacturer’s recommendations and at least one booster vaccination 15 months prior to our experiment. For this study, jugular blood was taken 14 days after booster vaccination with PregSure®BVD. After sampling, blood was immediately transferred to PAX gene blood RNA tubes (PreAnalytiX, Hombrechtikon, Switzerland). Samples were frozen and stored at −80 °C according to the manufacturer’s instructions until further processing. In addition to the whole blood from dams with divergent BNP phenotype, also the MDBK cell line was included. MDBK cells were grown in Eagle’s Minimal Essential Medium (EMEM) (Sigma-Aldrich Chemie, Steinheim, Germany) supplemented with 2 mM L-glutamine (Biochrom AG, Berlin, Germany), 1 % non-essential amino acids (NEAA) (Biochrom AG, Berlin, Germany) and 10 % heat-inactivated fetal calf serum (FCS) (PAN-Biotech GmbH, Aidenbach, Germany). Cells were maintained at 37 °C and 5 % CO_2_.

### Sample preparation

RNA from frozen whole blood samples was isolated with the PAXgene Blood RNA Kit (PreAnalytiX, Hombrechtikon, Switzerland). All procedures were performed according to the manufacturer’s instructions except for using twice the amount of RNase-free DNase I for on-column digestion of genomic DNA as recommended in the manufacturer’s instructions. Total RNA was prepared from the MDBK cell culture according to Demasius et al. [27]. RNA was stored at −80 °C until further processing. RNA concentration of all samples derived from whole blood cells and the MDBK cells was monitored on a Nanodrop ND-1000 system (Peqlab, Erlangen, Germany). RNA integrity was analyzed for all samples on a Bioanalyzer 2100 (Agilent, Böblingen, Germany). To assess whether the RNA samples were contaminated with genomic DNA, PCRs with genomic primers were carried out according to Weikard et al. [28]. In case of contamination with residues of genomic DNA, samples were treated with DNase I according to the RNAeasy MinElute Cleanup protocol (Qiagen, Hilden, Germany) until no traces of genomic DNA could be detected.

### Library preparation and deep sequencing

Library preparation and paired-end sequencing was essentially performed as described in Demasius et al. [27]. A multiplexed paired-end 61 cycle sequencing run on a Genome Analyzer GA IIx (Illumina, San Diego, USA) yielded the short paired-end reads used for further analysis.

The resulting reads were demultiplexed using CASAVA v 1.8 (https://support.illumina.com/sequencing/sequencing_software/casava.html). The demultiplexed reads of one sample from the different mixes and flow cells were merged into a single fastq file and checked for quality (base quality scores, adaptor contamination, repetitive sequences) using FastQC (http://www.bioinformatics.babraham.ac.uk/projects/fastqc/). The reads passing quality threshold served as input for further analyses.

### Catalogue of known classical and non-classical MHC class I sequences

Sequences of bovine classical and non-classical MHC class I alleles were obtained from the official *Bos taurus* Immuno Polymorphism Database (IPD-MHC BoLA webpage http://www.ebi.ac.uk/ipd/mhc/bola/index.html, accessed 2015/09/11) [29]. The sequence files of all classical and non-classical alleles were merged into a single data file, which was indexed for further sequence alignment using Samtools [30]. Classical and non-classical MHC class I alleles were combined into one file due to partial sequence identity and because phylogenetic analysis of MHC class Ia and Ib sequences revealed that classical MHC I genes and the non-classical MHC class I gene NC1 share a common ancestor [6].

### Sequence alignment

Alignment of reads obtained from deep sequencing was performed using Bowtie options (version 0.12.7) [31]. The reference sequence used for initial alignment comprised the catalogue of all known *Bos taurus* classical and non-classical MHC class I alleles.

**Fig. 1:**
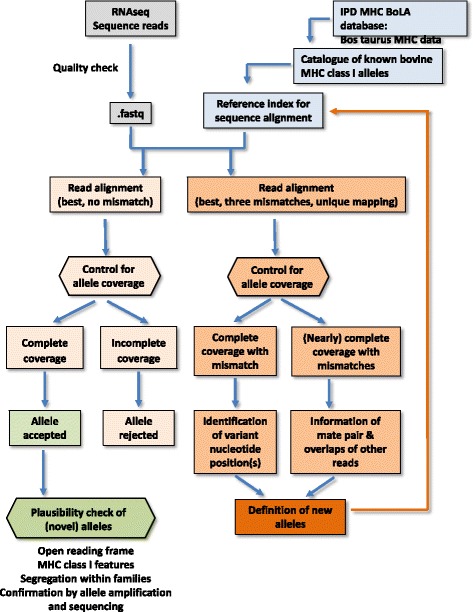
Workflow of RNAseq-assisted MHC class I typing (RAMHCIT)

For identifying the MHC class I alleles expressed by each of the individuals, we applied a stepwise approach within each sample (Fig. 1, Additional file 2). Initially, a very conservative alignment was conducted to distinguish the multiple MHC alleles with their high sequence diversity. We started with alignment to alleles in the initial MHC class I catalogue and accepted only those aligned reads that had no mismatch to the sequence of the respective alleles (Bowtie option –v0). For classifying an MHC class I allele to be present in an individual, the entire sequence of the respective allele had to be completely covered with reads (Additional file 3, A). This step of the typing process should identify all alleles represented in the catalogue of classical and non-classical alleles in the IPD-MHC database. However, due to the limited number of documented MHC class I alleles compared e.g., to human, it had to be assumed that novel, yet un-described alleles would be present in our data set. Thus, in addition to the non-mismatch tolerance procedure, a second alignment step was added for which we applied a relaxed stringency for sequence alignment allowing for up to three mismatches to the class I allele sequence library per read (Bowtie option –v3) with the option of obtaining only the best alignment for each read (Bowtie option --best) (Additional file 3, B). Due to their higher expected variability, the identification of novel alleles started with the classical MHC class I genes. For sequence detection of novel alleles, first those alleles were investigated that were fully covered with reads after relaxed alignment threshold or were fully covered except a single region < 5 bp. The resulting BAM file from sequence alignments was visually inspected for variant nucleotides compared to the “parent” allele by using the Integrative Genomics Viewer (IGV, [32]) in order to reveal the novel variant alleles. The newly discovered alleles show high sequence similarity to known “parent” alleles were then added to the catalogue of MHC class I alleles and the alignment procedure was repeated until no further new alleles were discovered. For detection of new alleles, we also visually inspected the BAM files for repetitively occurring unique mutations compared to all other identified alleles. Starting from the respective reads, we followed up the mate pair and matching sequence overlaps from the other reads of the same individuals to read the nucleotide sequence of the novel allele directly from the assembled reads. The generated sequence was also included into our MHC class I allele catalogue and tested for complete coverage in a final read alignment. For quantification of reads aligned to the final MHC class I allele catalogue, for each individual a final alignment was performed providing as reference sequence for alignment only those alleles that had been identified in that individual. Read counts per allele were then determined applying Unix commands (see Additional file 2).

After finishing the detection of novel alleles for classical MHC class I genes, the respective protocol as described above was repeated also for the analysis of the non-classical MHC class I genes. The reference sequence file for alignment contained all MHC class I alleles from the database and all newly discovered classical MHC class I alleles from this study.

Data files for MHC class I classical and non-classical genotyping for all individuals included in the study are deposited at the European Nucleotide Archive (http://www.ebi.ac.uk/ena/browse) under project number PRJEB12943.

### Allele validation by haplotype tracking using SNP data

For the eight F_2_ full sib animals, MHC class I haplotypes were manually derived from MHC class I genotyping data and compared to haplotypes established in previous studies (as indicated on the IPD-MHC BoLA webpage http://www.ebi.ac.uk/ipd/mhc/bola/index.html). To evaluate, whether these genotypes and haplotypes were correctly assigned, we conducted independent haplotype tracking from SNP data and checked genotypes and haplotypes for accordance with Mendelian inheritance. For this purpose, all F_2_ cows included in this study and also their F_1_ parents were genotyped with the 50 K bovine SNP chip (Illumina, San Diego, USA). This enabled definition and tracking of SNP haplotypes within and around the genomic MHC class I region (located at 28.3–28.5 Mb on *Bos taurus* chromosome 23 (BTA23) in the UMD 3.1 *Bos taurus* genome assembly [33]). For this purpose, all SNPs within the respective genomic region which included seven SNPs (BTA23 27,545,231 bp to 30,222,836 bp framed by SNPs rs110260956 and rs109862194) that had passed quality control (call rate >0.98, minor allele frequency >0.05, p(HWE) > 0.001) were filtered. SNPs heterozygous for at least one parent were individually screened for allelic inheritance in the offspring. As a consequence, pedigree-derived maternal and paternal haplotypes for the target region BTA23 27,545,231 bp to 30,222,836 bp) for all F_2_ full-sib cows in our data set could be established. In addition, paternally inherited haplotypes were analogously derived for the half sib individuals.

### Allele validation by Sanger sequencing

Sanger sequencing is commonly used as gold standard to evaluate novel immunogenotyping methods, also when based on whole genome sequencing (e.g., [34]). We used two different universal primer pairs and a set of specific oligonucleotides based on multiple alignment of the respective allele sequences as obtained from the IPD data base (in case of previously known alleles) or from this study (in case of new alleles) to amplify and sequence exon 2 and 3 from the bovine MHC class I genes to confirm the alleles initially reported by RNAseq in an independent Sanger Sequencing approach from genomic DNA. Sequences and direction of primers are given in Additional file 4 and their location is depicted in Additional file 5. For the analysis, genomic DNA from two individuals with a large number of novel and divergent alleles was extracted from leukocytes collected from whole blood by hypotonic lysis of erythrocytes. PCR cycling was done with a drop-down protocol for 15 min at 95 °C, and 9 cycles (30 s at 95 °C, 45 s at 62 °C–0.5 °C/cycle, 120 s at 74 °C), and 30 cycles (30 s at 94 °C, 45 s at 56 °C, 120 s at 74 °C) from 30 ng of DNA with standard buffer conditions, (2.0 mM MgCl_2_, dNTP’s, 25 nM each), and 0.35 units of HotStar-taq polymerase (Qiagen, Hilden, Germany) in a final volume of 20 μl on a 96-well thermocycler (Biometra, Göttingen, Germany). Primer concentrations were 300 nM. PCR products were sequenced using the BigDye® terminator v3.1 cycle sequencing kit (Life Technologies). The reactions were run on an ABI 3130 automated DNA sequencer and analyzed with the SeqScape™ software v2.7 (Applied Biosystems, Foster City, CA, USA).

### Analysis of amino acid sequences for MHC class I alleles

Nucleotide sequences of all new classical and non-classical MHC class I alleles were translated into predicted amino acid sequences, tested for open reading frames and characteristic MHC class I features. Subsequently, all MHC class I alleles were aligned using ClustalW Multiple Alignment in the BioEdit Sequence Alignment Editor (version 7.0.5.3.) ([35] http://www.mbio.ncsu.edu/bioedit/bioedit.html). All domains of the predicted amino acid sequences (according to [36, 37]) were inspected for differences or common features in the amino acid sequence between the MDBK cell line and the eight cows from the two BNP groups or the three cows from the control group. Furthermore, an analogous comparison between the three groups of cows with divergent BNP genotype was conducted.

## Results and discussion

### RNAseq data and alignment

For the whole blood samples from the 12 cows, paired-end sequencing yielded, after demultiplexing and quality control, 33,543,345–44,636,963 paired-end fragments per sample (Additional file 1). For the MDBK cell line, a total amount of 105,851,548 paired-end fragments was obtained. Alignment of reads from the whole blood samples to the final sequence library for each individual containing only those MHC I alleles expressed by the respective individual resulted in 182,158–714,466 reads with reported alignments (0.47–1.63 % of overall reads that could be aligned to the given alleles) (Additional file 1). 33,850 reads derived from the MDBK cell line (0.03 % of total reads) could be mapped to the final library of classical and non-classical MHC I sequences.

### Identification of classical MHC class I alleles

**Fig. 2:**
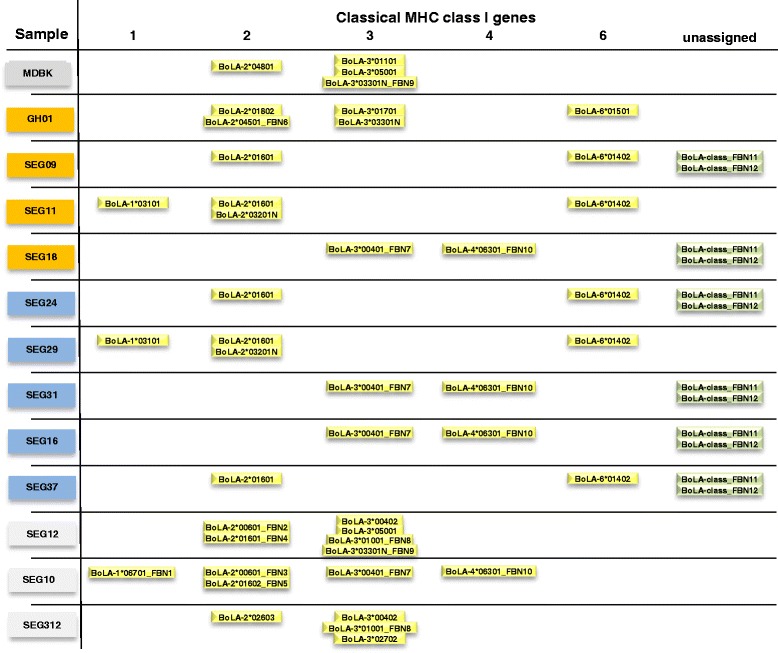
Overview of classical MHC class I alleles in the data set. Overview of previously published and novel classical MHC class I alleles identified in each sample/individual of the data set. MDBK: Madin-Darby bovine kidney cells; GH, German Holstein; orange box: cow which had calves with clinical BNP; blue boxes: cow which had calves showing no clinical BNP, but hematological deviations; grey box: control cow; yellow boxes: MHC class I alleles assigned to classical MHC class I genes; green boxes: *de novo* derived MHC class I alleles unassigned to MHC class I genes

All assignments of alleles from the IPD-MHC BoLA data base to specific classical MHC class I genes were made according to the classification described in Hammond et al. [38]. Although 11 of the cows investigated in this study belonged to an eight-individual fullsib or a three-individual half sib family, respectively, our novel method of comprehensive MHC class I typing revealed substantial allele diversity. The initial analysis based on the alleles in the IPD-MHC BoLA database identified a total of 12 alleles (Fig. 2): one allele for the MHC class I gene 1, four alleles for MHC class I gene 2, five allele for MHC class I gene 3, no alleles for MHC class I genes 4 and 5 and two alleles for MHC class I gene 6 (allele assignment according to http://www.ebi.ac.uk/ipd/mhc/bola/index.html and Codner et al. [6]) (Fig. 2). Those alleles were fully covered with reads after initial alignment to the catalogue of sequences from the IPD-MHC BoLA database. Subsequent sequence analysis of reads after alignments with relaxed threshold for mismatches identified further 12 alleles related to known alleles from four classical MHC class I genes in the blood transcriptome of the 12 cows (Fig. 2): One allele for gene 1, five alleles for gene 2 alleles, three alleles for gene 3, one allele for gene 4 and two alleles were de novo derived from direct read sequences (BoLA-FBN11, BoLA-FBN12) and could not be unequivocally assigned to a MHC class I gene. All new classical MHC class I alleles differing from previously described BoLA alleles at nucleotide level were also polymorphic at the predicted amino acid sequence level.

There were three groups of individuals in the F_2_ full-sib ship with identical MHC class I genotype. Cows SEG09, SEG24 and SEG37 expressed all alleles in common, while the second group sharing expressed alleles consisted of cow SEG11 and SEG29. Finally, cows SEG 31, SEG 16 and SEG 18 showed identical MHC class I genotypes.

**Fig. 3:**
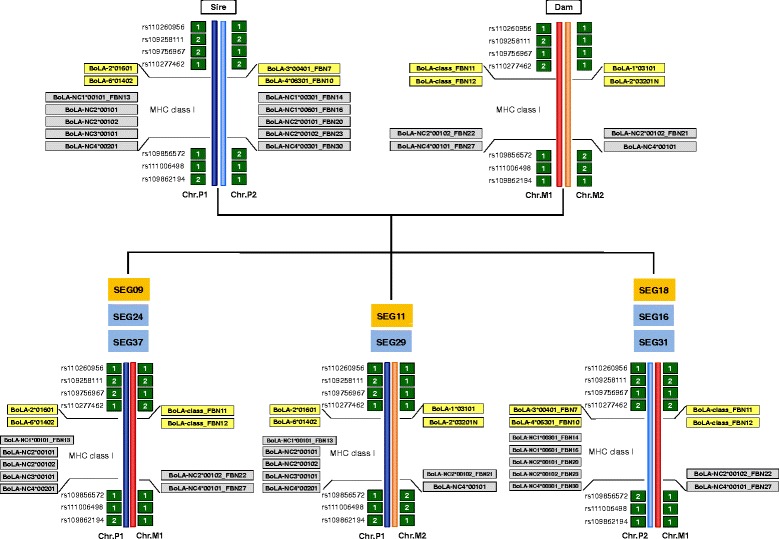
MHC class I and SNP haplotype tracking in a F_2_ full-sib family. Detection of paternally and maternally inherited MHC class I haplotypes. Identification of three groups with the same MHC class I genotypes. Comparison and confirmation of MHC class I haplotypes with available genotype data (SNP-data) for each individual. rs-number: reference SNP cluster ID; Chr.P1/Chr.P2: alternative paternal haplotypes; Chr.M1/Chr.M2: alternative maternal haplotypes; orange box: cow which had calves with clinical BNP; blue boxes: cow which had calves showing no clinical BNP, but hematological deviations; green boxes: SNP alleles; yellow boxes: classical MHC class I alleles; grey boxes: non-classical MHC class I alleles; bluish strands: paternal chromatids; reddish strands: maternal chromatids. According to the Immuno Polymorphism Database (IPD-MHC; www.ebi.ac.uk/ipd/mhc/bola) haplotype 2*1601–6*1402 corresponds to haplotype A19, haplotype 1*03101–2*03201N corresponds to haplotype A13, haplotype 3*00402–3*01001_FBN8 is a variant of haplotype A2/A30

The eight F_2_-full sibs in our data set enabled detection of maternally and paternally inherited MHC class I haplotypes (Fig. 3). Two of the classical MHC class I haplotypes correspond to previously established haplotypes (A13: 1*03101–2*03201N, A19: 2*1601–6*1402, http://www.ebi.ac.uk/ipd/mhc/bola/index.html, IPD-MHC BoLA database). The respective haplotypes had been described already for the Holstein breed, a founder breed of our F_2_ cross population. All identified MHC class I haplotypes were also compared to haplotype tracking results based on 50 K genotyping in the MHC class I genomic region (Fig. 3, Additional file 6). Allele and haplotype tracking of MHC class I and SNP alleles were in full agreement with Mendelian inheritance of all alleles identified in this study. This applied not only to the alleles from the IPD-MHC BoLA data base, but also to all new alleles identified.

### Identification of non-classical MHC class I alleles

**Fig. 4:**
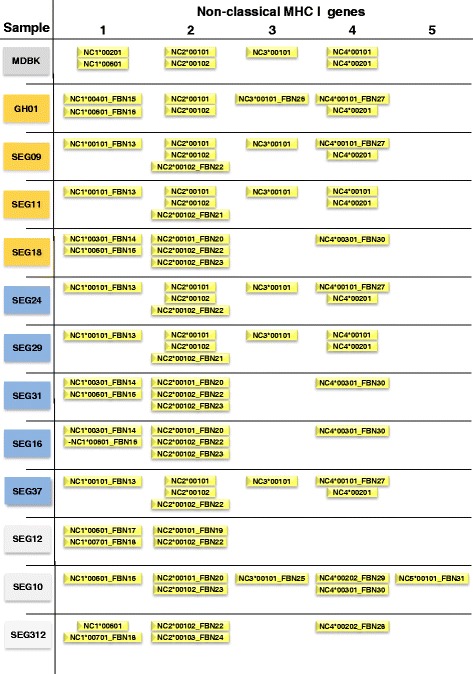
Overview of non-classical MHC class I alleles in the data set. Overview of previously published and novel non-classical MHC class I alleles identified in each sample/individual of the data set. MDBK: Madin-Darby bovine kidney cells; GH, German Holstein; orange box: cow which had calves with clinical BNP; blue boxes: cow which had calves showing no clinical BNP, but hematological deviations; grey box: control cow; yellow boxes: MHC class I alleles assigned to non-classical MHC class I genes

Initial data analysis with the list of non-classical MHC class I alleles from the IPD-MHC BoLA database identified a total of 6 previously described alleles in our data set (Fig. 4): one allele for NC1 and NC3, respectively, and two alleles for NC2 and NC4, respectively. No NC5 allele was detected. Subsequently, a total of further 19 alleles with non-classical MHC class I structural allele features were discovered: six alleles with high sequence similarity to NC1 and NC2, respectively, two alleles very similar to NC3, four alleles to NC4 and one allele to NC5. Five individuals did not express NC3 alleles and in one of them (SEG12) also no NC4 allele was identified. All novel derived non-classical alleles display characteristic features of non-classical MHC class I alleles like an early stop codon or a VPI, IPI or VLIK motif [39] in the transmembrane domain. Analogous to the classical MHC class I alleles, analysis of segregation pattern of haplotypes within full and half sibship was in agreement with Mendelian laws of inheritance (Fig. 3, Additional file 6). All eight F_2_ full sib individuals showed three alleles supposed to originate from NC2 suggesting a NC2 gene duplication event (Fig. 4). This was confirmed by haplotype tracking within the pedigree suggesting two paternal haplotypes both carrying two NC2 copies (Fig. 3). Furthermore, gene duplication for NC1 was discovered by haplotype tracking for one paternal haplotype.

### Quantification of MHC class I allele expression

An overview of the number of reads mapping to each classical and non-classical MHC class I allele expressed by each individual is given in Tables 1 and 2. Except one allele (BoLA3*0331 N-FBN9), all other newly identified alleles showed expression levels within the range of those alleles from the IPD-MHC BoLA database. The low expression of allele BoLA3*0331 N-FBN9 is analogous to the BoLA3*0331 N allele, which is an established MHC class I null allele. The number of reads assigned to single alleles varies substantially within individual. However, across individuals the relative proportion of reads assigned to alleles is remarkably constant. Individuals sharing the same MHC class I alleles also exhibit nearly identical proportions of reads for the different alleles. This indicates that consistent unequal expression of alleles is present, which can be reliably detected by our RAMHCIT approach. In the past, essentially all studies on quantitative expression have been restricted to protein level [40]. They monitored artificial expression of MHC class I alleles in suitable MHC null cell lines by pan-MHC class I antibodies. Only recently, first attempts for MHC class I allele quantification using PCR-based next generation sequencing (NGS) technology have been published [40, 41]. However, due to abundant polymorphism and differing number of class I genes per haplotype, a reliable allele specific expression measurement is hardly feasible using conventional methods in species with complex MHC class I structures like bovine. Still, there are initial reports documenting unequal expression across MHC class I alleles in pigs applying PCR amplification-based NGS technology on a 454 Roche System [41]. In spite of MHC class I specific RNA amplification, which might have masked differences in allelic expression, Kita et al. [41] found that the percentage of allele-specific reads was very similar in different individuals and was about half in heterozygous compared to homozygous animals.

**Table 1:** Proportion of reads mapping to a specific classical allele relative to total reads mapping to all MHC class I classical alleles within each individual

MHC class I gene	Allele	Individual											
		GH01	SEG09	SEG11	SEG18	SEG24	SEG29	SEG31	SEG16	SEG37	SEG12	SEG10	SEG312
1	1\*03101			0.295			0.303						
	1\*06701_FBN_1											0.188	
2	2\*00601_FBN_2										0.110		
	2\*00601_FBN_3											0.177	
	2\*01601		0.172	0.209		0.180	0.205			0.173			
	2\*01601_FBN_4										0.168		
	2\*01602_FBN_5											0.151	
	2\*01802	0.251											
	2\*02603												0.251
	2\*03201N			0.131			0.119						
	2\*04501_FBN_6	0.262											
3	3\*00401_FBN_7				0.084			0.085	0.081			0.115	
	3\*00402										0.150		0.097
	3\*01001_FBN_8										0.296		0.178
	3\*01701	0.170											
	3\*02702												0.474
	3\*03301N	0.012											
	3\*03301N_FBN_9										0.016		
	3\*05001										0.260		
4	4\*06301_FBN_10				0.232			0.235	0.233			0.369	
6	6\*01402		0.317	0.365		0.311	0.372			0.319			
	6\*01501	0.305											
Un-assigned	class_FBN_11		0.317		0.422	0.317		0.415	0.421	0.322			
	class_FBN_12		0.194		0.262	0.192		0.264	0.265	0.186

**Table 2:** Proportion of reads mapping to a specific non-classical allele relative to total reads mapping to all MHC class I non-classical alleles within individual

MHC class I gene	Allele	Individual											
		GH	SEG09	SEG11	SEG18	SEG24	SEG29	SEG31	SEG16	SEG37	SEG12	SEG10	SEG312
NC 1	NC1\*00101_FBN_13		0.128	0.072		0.149	0.075			0.114			
	NC1\*00301_FBN_14				0.110			0.112	0.102				
	NC1\*00401_FBN_15	0.087											
	NC1\*00601												0.323
	NC1\*00601_FBN_16	0.108			0.098			0.104	0.100			0.117	
	NC1\*00601_FBN_17										0.280		
	NC1\00701_FBN_18										0.075		0.122
NC 2	NC2\*00101	0.261	0.200	0.168		0.177	0.163			0.186			
	NC2\*00101_FBN_19										0.340		
	NC2\*00101_FBN_20				0.194			0.190	0.199			0.146	
	NC2\*00102	0.293	0.175	0.135		0.157	0.139			0.166			
	NC2\*00102_FBN_21			0.236			0.222						
	NC2\*00102_FBN_22		0.247		0.273	0.226		0.269	0.254	0.240	0.306		0.358
	NC2\*00102_FBN_23				0.224			0.220	0.241			0.185	
	NC2\*00103_FBN_24												0.127
NC 3	NC3\*00101		0.222	0.370		0.262	0.384			0.248			
	NC3\*00101_FBN_25											0.281	
	NC3\*00101_FBN_26	0.199											
NC 4	NC4\*00101			0.012			0.011						
	NC4\*00101_FBN_27	0.026	0.018			0.019				0.026			
	NC4\*00201	0.026	0.011	0.007		0.011	0.007			0.020			
	NC4\*00202_FBN_28												0.071
	NC4\*00202_FBN_29											0.069	
	NC4\*00301_FBN_30				0.101			0.104	0.103			0.050	
NC 5	NC5\*00101_FBN_31											0.152	

### Identification of classical and non-classical MHC class I alleles in the MDBK cell line

Aligning reads to the classical MHC class I alleles from our extended IPD-MHC BoLA database yielded a complete coverage of the MHC class I alleles BoLA-2*04801, BoLA-3*01101 and BoLA-3*05001 for the MDBK cell line (Fig. 2). Applying conventional PCR-based Sanger sequencing, Bell et al. [25] and Benedictus et al. [42] also identified these alleles in the MDBK cell line, which confirms that RAMHCIT is able to reliably detect MHC class I alleles. According to Codner et al. [6], BoLA-3*03301N is very closely related to BoLA allele 3*00402. Bell et al. [25] reported a variant BoLA allele 3*00402v being present in the MDBK cell line. However, the authors did not reveal the specific sequence of their newly described allele, but it might be suggested that BoLA-3*03301N_FBN9 and BoLA allele 3*00402v share the same sequence. Alignment of the reads from the MDBK cell line resulted in a complete coverage for the non-classical MHC class I alleles BoLA-NC2*00101, BoLA-NC2*00102, BoLA-NC3*00101, BoLA-NC4*00101 and BoLA-NC4*00201. Although we sequenced the MDBK transcriptome to a very deep coverage (>100 million paired-end reads), we obtained only a low number of reads aligning to MHC class I (Additional file 1), and as a consequence no clear evidence for NC1*00201 and BoLA-NC1*00601 allele expression could be obtained. This is mainly due to the different cell type and indicates that an appropriate number of sequence reads has to be available for reliable allele detection. The number of total read counts for class I genes per transcriptome will depend on the tissue and physiological stage of the cells that express the target gene for MHC class I typing.

In principle, our method enables the detection of all expressed non classical and classical MHC class I genes. Since we could derive haplotypes from genotyping complete families some initial conclusions on the number of genes per haplotype could be drawn. In our limited set of animals, haplotypes carry 2–3 classical class I genes and 2–5 non classical class I genes.

### Confirmation of MHC class I alleles by locus specific experimental Sanger sequencing

Exons 2 and 3 of classical and non-classical MHC class I alleles were amplified and sequenced from genomic DNA for two animals to confirm the results from RAMHCIT. The alleles that were identified by RNAseq were selectively amplified and sequenced using a set of allele specific oligonucleotides. All alleles were successfully sequenced for SEG29 (alleles BoLA-2*01601, 6*01402, 1*03101, 2*03201N, BoLA-NC1*00101_FBN13, NC2*00101, NC2*00102, NC3*00101, NC4*00201, NC2*00102_FBN21, NC4*00101) as well as for SEG18 (alleles BoLA-3*00401_FBN7, 4*06301_FBN10, FBN11, FBN12, BoLA-NC1*00301_FBN14, NC1*00601_FBN16, NC2*00101_FBN20, NC2*00102_FBN23, NC4*00301_FBN30, NC2*00102_FBN22).

### Evaluating MHC class I alleles for mono-causal background of BNP

The clustering of cows in the F_2_ full-sib family into three groups according to MHC class I allele genotypes did not correspond to their classification regarding BNP status. All three MHC class I genotype groups comprised individuals from the BNP-C and BNP-H group (Figs. 2 and 4). Furthermore, according to the hypothesis of distinct MHC class I alleles being causal for BNP, the control cows should share alleles with the MDBK cells that are not present in BNP cows. Comparison of classical MHC class I alleles between BNP-H/BNP-C and the control group showed that control cows expressed 11 alleles that were not detected in BNP-H and BNP-C cows (Fig. 2). For non-classical MHC class I genes, control cows expressed 9 alleles, which were all absent in the BNP-H or BNP-C group. These 20 alleles would represent potential candidates that might be involved in BNP aetiopathology. However, when comparing the list of alleles exclusively expressed in cows from the control group, which had not produced BNP colostrum, only one of those alleles is expressed in the MDBK cells. Looking at the MDBK alleles, only MDBK allele BoLA-3*05001 is also expressed in one of the control cows (control cow SEG12).

Comparison of MHC class I allele sharing between cows with divergent BNP status and MDBK cells at nucleotide level yielded no indication on potential causal alleles for BNP. However, production of potential causal BNP antibodies depends on the particular epitopes of the MHC class I alleles. Thus, the comparison of allele sharing was extended to amino acid level. We found no position in the classical MHC class I alleles of the MDBK cells or the control cows, where the amino acid differed to the respective position in all BNP-C cow alleles (Additional file 7). Analogously, no amino acid position of a specific non-classical MHC class I allele observed in the MDBK cell line was shared by all control cows and was different in all non-classical alleles of BNP-C cows (Additional file 8). Moreover, since the MDBK cell line shared all fully covered non-classical MHC class I alleles (BoLA-NC2*00101, BoLA-NC2*00102, BoLA-NC3*00101, BoLA-NC4*00101, and BoLA-NC4*00201) with one dam of group BNP-C and one dam of group BNP-H, these alleles could already be excluded as single causal alleles for BNP.

From these data at nucleotide resolution level, no specific target BNP MHC class I allele representing a potential monocausal background for BNP could be detected. This confirms recent studies reporting antibody binding of BNP serum to a large variety of MHC class I alleles and not restricted to alleles expressed in the MDBK cell line [25].

### MHC typing applying deep RNAseq as a new tool for identifying previously unknown MHC alleles

Due to the high demand for a comprehensive MHC class I genotyping method in many applications from clinical medicine to evolutionary studies, several new approaches beyond traditional serological or single target PCR techniques have been described. These include whole genome/transcriptome read alignment to a catalogue of known MHC class I alleles [11, 12], single-position variant calling from whole genome sequence reads relative to the species genome assembly [34], *de novo* assembly of MHC class I alleles from whole genome sequence reads [13], PCR pre-amplification combined with Sanger or 454 pyrosequencing [10, 43–45] or a combination of methods [46]. For HLA typing in humans, different sequencing and typing software have been applied, which are reviewed in [47].

For our new RAMHCIT method, Sanger sequencing of alleles and allele/haplotype tracking confirmed not only those alleles that had been previously described. RAMHCIT was also able to identify a substantial number of new alleles for classical and non-classical MHC class I genes, which are related to previously described alleles, and even two completely new alleles established exclusively from RNAseq data without specific parental allele. In-silico analysis of the predicted amino acid sequence of those alleles indicated that all new alleles should be functional (e.g., due to open reading frame or structural features typical for MHC class I alleles). This demonstrates the capacity of the RAMHCIT approach to identify nearly all expressed alleles of an individual and to add new alleles to a currently incomplete BoLA allele catalogue. Thus, RAMHCIT is especially useful for populations and breeds that had not been characterized comprehensively.

Compared to the previously described methods, RAMHCIT represents an untargeted approach that enables the unbiased detection of all expressed alleles and is not restricted to detection of alleles, which had been successfully amplified in an initial PCR step or are included in comprehensive MHC class I catalogues. In contrast, PCR-based methods are inherently subject to the risk of missing alleles that are polymorphic at the primer binding sites, because they rely on gene-specific PCR amplification of cDNA or genomic DNA using gene-specific primers and the subsequent analysis of those gene-specific PCR products using either Sanger sequencing [44] or NGS platforms as has been demonstrated in other studies [10, 43, 48].

Moreover, RAMHCIT is not restricted to the existing bovine reference genome assembly, which per definition can only represent a single specific MHC class I haplotype. Instead, RAMHCIT exploits a catalogue containing all currently deposited MHC class I alleles for sequence alignment. In humans, Huang et al. [13] demonstrated that mapping NGS reads exclusively to a reference genome assembly resulted in loosing read alignments and subsequently in incomplete HLA typing. In species, where individuals differ not only in allelic structure, but also in the identity and number of MHC class I genes, this peril to reliable genotyping is even higher. This applies e.g., to non-human primates [49] or to livestock species e.g., cattle [6]. Our method alleviated this problem, because starting from a given list of alleles it enables stepwise extending this list during the genotyping process. The complex polymorphic structure of the MHC class I locus in some species also hampers approaches including exclusively *de novo* assembly of reads as implemented e.g., in the HLAreporter typing tool [13]. In cattle, the problem with *de novo* assembly is further acerbated by the strong sequence similarity between genes [38]. Dudley et al. [46] developed an RNAseq-based method combining initial read alignment to known alleles with subsequent extension/*de-novo* assembly guided by the allele sequences obtained in the first step. However, in contrast to RAMHCIT, the method from Dudley at al. [46] relies on two PCR pre-amplification steps, which carries the limitations of all PCR-based MHC class I genotyping methods (see above).

Due to the fact that allele calling in RAMHCIT requires full sequence read coverage for a respective allele for allele calling, the problem of preference for calling the reference allele encountered by standard SNP detection tools [34] is avoided in RAMHCIT. RAMHCIT also is not dependent on population data regarding allelic frequencies of MHC class I alleles, which are part of the analysis pipeline of other MHC genotyping tools, e.g., HLAreporter [13], and, thus, RAMHCIT is applicable also to poorly investigated breeds/populations.

Finally RAMHCIT also opens up the perspective to analyze simultaneously allele identity and the relative expression levels of MHC class I alleles, which is not feasible for PCR based methods due to different allele amplification efficiencies of primers during data processing [10].

## Conclusions

In conclusion, our study has provided evidence that specific classical and non-classical MHC class I can be excluded as single causal agents for BNP-associated alloantibodies using a new approach combining deep sequencing transcriptome analysis by RNAseq and a novel MHC typing strategy. This new methodological procedure RAMHCIT has enabled detection of several previously unknown classical and non-classical MHC class I sequences, providing the opportunity to add more information to the IPD-MHC BoLA database. Since the class I family is more complex than class II and III in cattle, it can be expected, that this method will work for bovine class II genes as well. The newly developed method is a novel innovative technique for high-resolution MHC class I genotyping in non-model species. RAMHCIT can be applied to non-model species with limited information on MHC allele diversity for high-resolution MHC class I genotyping as required for analyses of immune response, for clinical applications and evolutionary studies.

### Accession numbers

Sequences of all 31 new classical and non-classical bovine MHC class I alleles were deposited in GenBank under following accession numbers: BoLA-1*06701_FBN1: KT428346, BoLA-2*00601_FBN2: KT428347, BoLA-2*00601_FBN3: KT428348, BoLA-2*01601_FBN4: KT428349, BoLA-2*01602_FBN5: KT428350, BoLA-2*04501_FBN6: KT428351, BoLA-3*00401_FBN7: KT428352, BoLA-3*01001_FBN8: KT428353, BoLA-3*03301N_FBN9: KT428354, BoLA-4*06301_FBN10: KT428355, BoLA_UN_FBN11: KT428356, BoLA_UN_FBN12: KT428357, BoLA-NC1*00101_FBN13: KT428358, BoLA-NC1*00301_FBN14: KT428359, BoLA-NC1*00401_FBN15: KT428360, BoLA-NC1*00601_FBN16: KT428361, BoLA-NC1*00601_FBN17: KT428362, BoLA-NC1*00701_FBN18: KT428363, BoLA-NC2*00101_FBN19: KT428364, BoLA-NC2*00101_FBN20: KT428365, BoLA-NC2*00102_FBN21: KT428366, BoLA-NC2*00102_FBN22: KT428367, BoLA-NC2*00102_FBN23: KT428368, BoLA-NC2*00103_FBN24: KT428369, BoLA-NC3*00101_FBN25: KT428370, BoLA-NC3*00101_FBN26: KT428371, BoLA-NC4*00101_FBN27: KT428372, BoLA-NC4*00202_FBN28: KT428373, BoLA-NC4*00202_FBN29: KT428374, BoLA-NC4*00301_FBN30: KT428375, BoLA-NC5*00101_FBN31: KT428376.

## Additional files

Additional file 1: Table S1. Overview of samples included in the analysis regarding BNP status and statistics of read alignments. (DOCX 19 kb) Additional file 2: Workflow S1. Description of bioinformatic data analysis of transcriptomic RNAseq data for MHC class I genotyping. Bioinformatic skripts including codes and options for preprocessing and data analysis of fastq files from RNAseq transcriptome analysis for MHC class I allele identification and genotyping. (ZIP 9 kb) Additional file 3: Figure S1. Examples of read coverage for single MHC class I alleles in individual cows. A: Read coverage of the allele BoLA-2*1601 in the full-sibs SEG11 and SEG31. SEG11 shows complete coverage of reads with the option of zero mismatches (0mm) per read. SEG31 displays incomplete coverage of reads even with the option of three mismatches (3mm) per read indicating that the allele BoLA-2*1601 is not expressed in this animal. B: Read coverage of the previously published allele BoLA-2*00601 in cow SEG12. Alignment allowing for three (3mm) or zero mismatches (0mm) per read in order to identify novel variant alleles. (PDF 220 kb) Additional file 4: Table S2. Allele specific primers used for experimental MHC class I allele confirmation by Sanger sequencing. The table lists the oligonucleotide primers used for amplification and sequencing of MHC class I sequences. (DOCX 16 kb) Additional file 5: Figure S2. Positions of primers used for MHC class I allele amplification and sequencing. For primer sequences see Additional file 4 (Table S2). (PDF 121 kb) Additional file 6: Figure S3. MHC class I and SNP haplotype tracking in a F_2_ full-sib family. Detection of paternally and maternally inherited MHC class I haplotypes. Bluish strands: paternal chromatids; reddish strands: maternal chromatids; rs-number: Reference SNP cluster ID; green boxes: SNP alleles; yellow boxes: classical MHC class I alleles; grey boxes: non-classical MHC class I alleles; alleles deduced from genotypes of progeny are indicated in italic. (PDF 286 kb) Additional file 7: Figure S4. Classical MHC class I alleles: multiple alignment of amino acids. Alignment of the predicted amino acid sequences for classical MHC class I derived from the nucleotide sequence of alleles expressed in the data set. Dots: indicate identity to the first allele in the list; dashes: represent gaps compared to the first allele in the list; asterisk: denotes a stop codon; yellow labelled amino acids: uniquely occurring amino acid in an allele at a specific amino acid position in comparison to the other alleles. (DOCX 34 kb) Additional file 8: Figure S5. Non-classical MHC class I alleles: multiple alignment of amino acids. Alignment of the predicted amino acid sequences for classical MHC class I derived from the nucleotide sequence of alleles expressed in the data set. Dots: indicate identity to the first allele in the list; dashes: represent gaps compared to the first allele in the list; asterisk: denotes a stop codon. (DOCX 33 kb)

## Abbreviations

BAM file: binary alignment/map file
BNP: bovine neonatal pancytopenia
BoLA: Bovine Leukocyte Antigen
BTA: *Bos taurus* chromosome
BVD: Bovine Virus Diarrhea
EMEM: Eagle’s Minimal Essential Medium
FCS: fetal calf serum
HLA: Human Leukocyte Antigen
IGV: Integrative Genomics Viewer
IPD: Immuno Polymorphism Database
MDBK: Madin-Darby bovine kidney cell line
MHC: major histocompatibility complex
NC: non-classical MHC class I gene
NEAA: non-essential amino acids
RAMHCIT: RNAseq-assisted method for MHC typing
RNAseq: RNA sequencing
SNP: single nucleotide polymorphism
