# Supplementary material for: A novel RNAseq–assisted method for MHC class I genotyping in a non-model species applied to a lethal vaccination-induced alloimmune disease
Source: BMC Genomics. 2016 May 17;17:365. doi: 10.1186/s12864-016-2688-0 (PMC4869273; doi:10.1186/s12864-016-2688-0)
Supplement: Additional file 1: Table S1. — Overview of samples included in the analysis regarding BNP status and statistics of read alignments. (DOCX 19 kb) [file 12864_2016_2688_MOESM1_ESM.docx]

**Additional file 1 Table S1. Overview of samples included in the analysis regarding BNP status and statistics of read alignments**

| Sample | Breed | BNP status | Total number of paired-end reads | Reads aligned to classical MHC class I alleles | Reads aligned to non-classical MHC class I alleles | Reads aligned to all MHC class I alleles | % of all reads aligned to MHC class I alleles |
| --- | --- | --- | --- | --- | --- | --- | --- |
| MDBK |  |  | 105,851,548 | 26,734 | 7,116 | 33,850 | 0.03 |
| GH01 | GH | BNP-C | 33,543,345 | 318,772 | 31,422 | 350,194 | 1.04 |
| SEG09 | Cha x GH F_2_ | BNP-C | 44,627,365 | 520,032 | 45,342 | 565,374 | 1.27 |
| SEG11 | Cha x GH F_2_ | BNP-C | 44,636,963 | 469,820 | 59,764 | 529,584 | 1.19 |
| SEG18 | Cha x GH F_2_ | BNP-C | 39,030,040 | 165,000 | 17,158 | 182,158 | 0.47 |
| SEG24 | Cha x GH F_2_ | BNP-H | 41,569,788 | 437,068 | 37,744 | 474,812 | 1.14 |
| SEG29 | Cha x GH F_2_ | BNP-H | 44,155,016 | 447,330 | 56,178 | 503,508 | 1.14 |
| SEG31 | Cha x GH F_2_ | BNP-H | 37,375,624 | 312,960 | 29,886 | 342,846 | 0.92 |
| SEG16 | Cha x GH F_2_ | BNP-H | 37,819,325 | 331,178 | 31,404 | 362,582 | 0.96 |
| SEG37 | Cha x GH F_2_ | BNP-H | 38,731,113 | 446,462 | 51,510 | 497,972 | 1.29 |
| SEG12 | Cha x GH F_2_ | Control | 38,024,182 | 323,730 | 36,476 | 360,206 | 0.95 |
| SEG10 | Cha x GH F_2_ | Control | 39,502,827 | 258,004 | 42,890 | 300,894 | 0.76 |
| SEG312 | Cha x GH F_2_ | Control | 43,718,688 | 682,798 | 31,668 | 714,466 | 1.63 |

MDBK: Madin-Darby bovine kidney cells; GH: German Holstein; Cha: Charolais; BNP-C: Cow with calves displaying clinical bovine neonatal pancytopenia. BNP-H: Cow with calves displaying no clinical bovine neonatal pancytopenia but hematological deviations from the average of the peer group, cows were full sibs to SEG09, SEG11 and SEG18. Control: Cows from sire lines unaffected by BNP and with calves lacking any clinical or hematological indications on BNP
