# Supplementary figures and images for: A novel RNAseq–assisted method for MHC class I genotyping in a non-model species applied to a lethal vaccination-induced alloimmune disease
Source: BMC Genomics. 2016 May 17;17:365. doi: 10.1186/s12864-016-2688-0 (PMC4869273; doi:10.1186/s12864-016-2688-0)

A

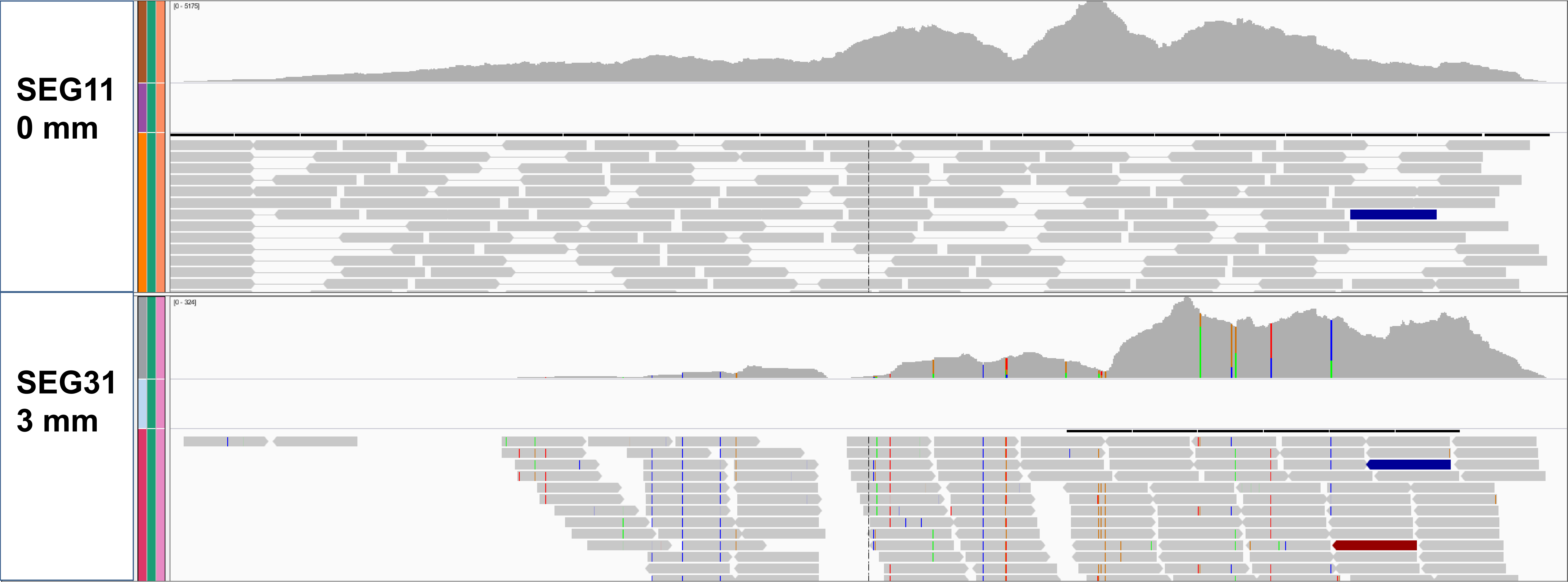

B

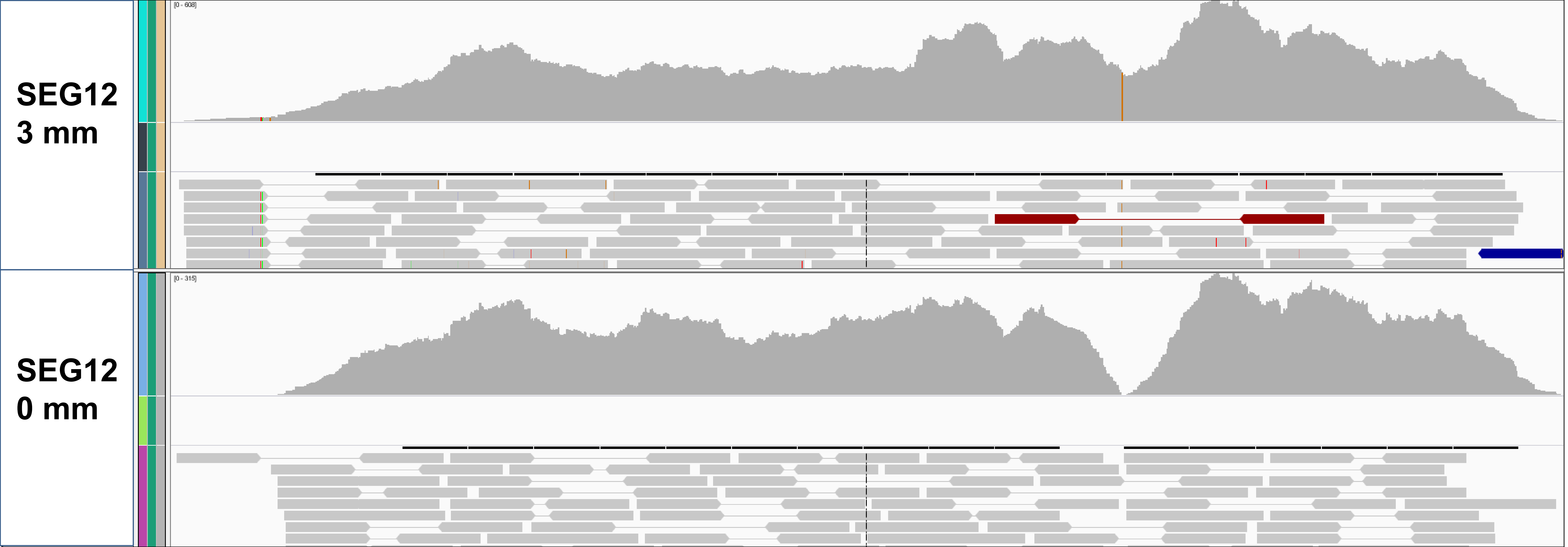

Supplement: Additional file 3: Figure S1. — Examples of read coverage for single MHC class I alleles in individual cows. A: Read coverage of the allele BoLA-2*1601 in the full-sibs SEG11 and SEG31. SEG11 shows complete coverage of reads with the option of zero mismatches (0mm) per read. SEG31 displays incomplete coverage of reads even with the option of three mismatches (3mm) per read indicating that the allele BoLA-2*1601 is not expressed in this animal. B: Read coverage of the previously published allele BoLA-2*00601 in cow SEG12. Alignment allowing for three (3mm) or zero mismatches (0mm) per read in order to identify novel variant alleles. (PDF 220 kb) [file 12864_2016_2688_MOESM3_ESM.pdf]

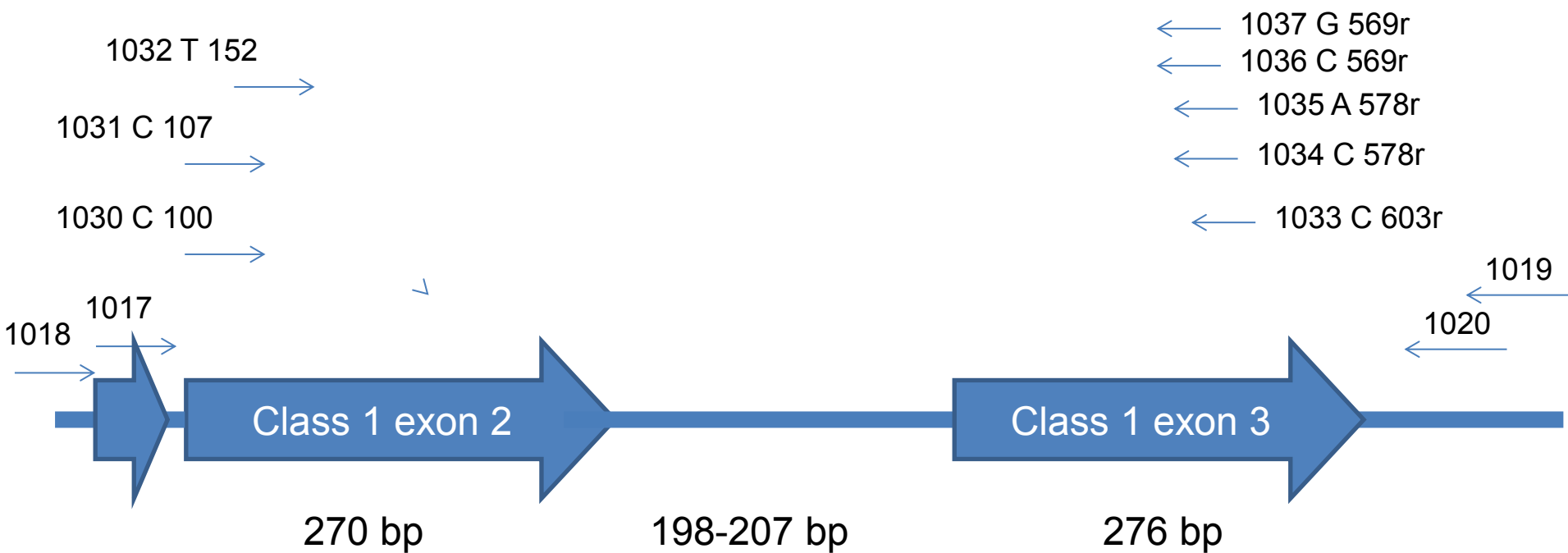

Supplement: Additional file 5: Figure S2. — Positions of primers used for MHC class I allele amplification and sequencing. For primer sequences see Additional file 4 (Table S2). (PDF 121 kb) [file 12864_2016_2688_MOESM5_ESM.pdf]

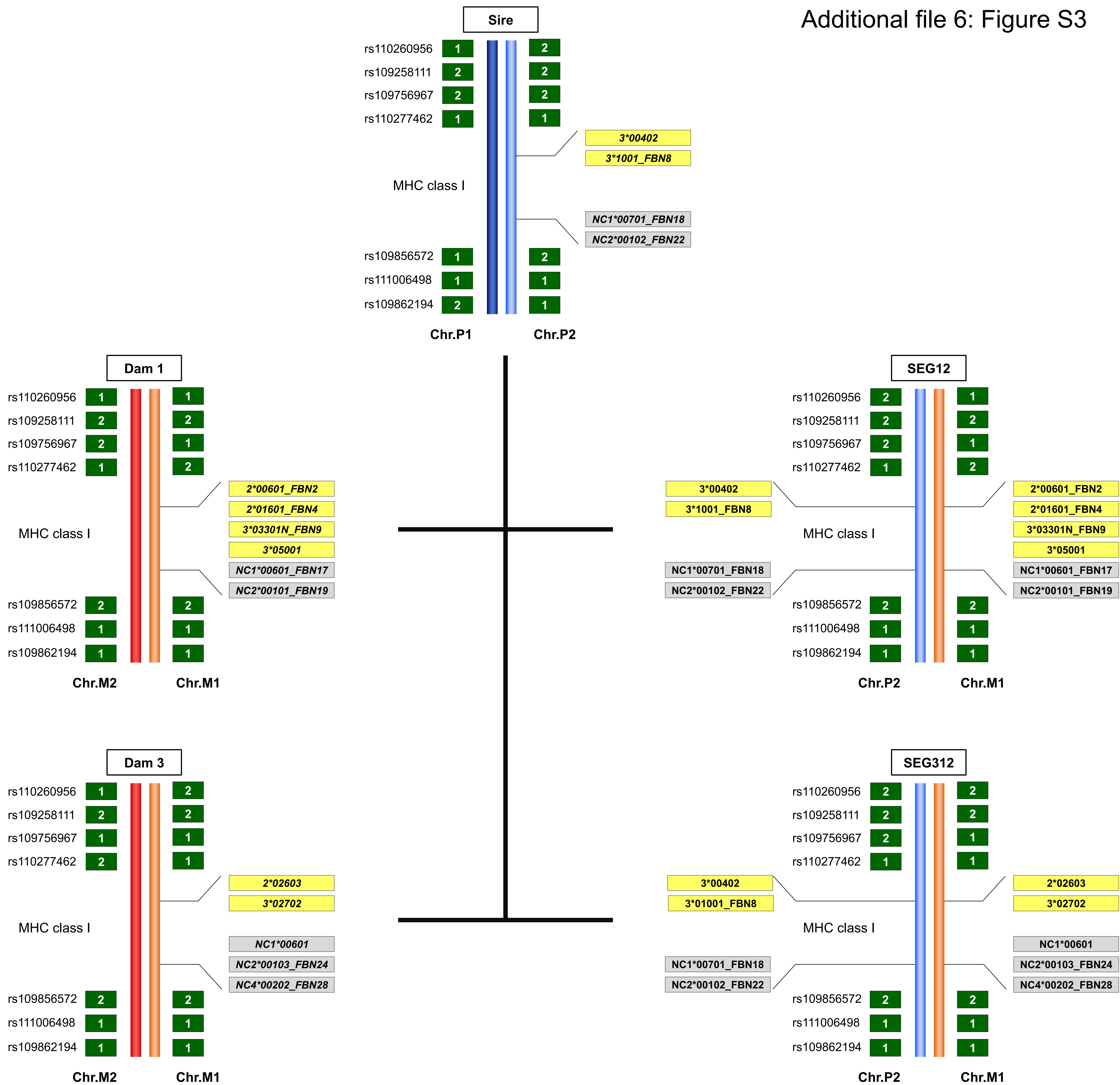

Supplement: Additional file 6: Figure S3. — MHC class I and SNP haplotype tracking in a F2 full-sib family. Detection of paternally and maternally inherited MHC class I haplotypes. Bluish strands: paternal chromatids; reddish strands: maternal chromatids; rs-number: Reference SNP cluster ID; green boxes: SNP alleles; yellow boxes: classical MHC class I alleles; grey boxes: non-classical MHC class I alleles; alleles deduced from genotypes of progeny are indicated in italic. (PDF 286 kb) [file 12864_2016_2688_MOESM6_ESM.pdf]
