# Supplementary material for: A novel RNAseq–assisted method for MHC class I genotyping in a non-model species applied to a lethal vaccination-induced alloimmune disease
Source: BMC Genomics. 2016 May 17;17:365. doi: 10.1186/s12864-016-2688-0 (PMC4869273; doi:10.1186/s12864-016-2688-0)
Supplement: Additional file 4: Table S2. — Allele specific primers used for experimental MHC class I allele confirmation by Sanger sequencing. The table lists the oligonucleotide primers used for amplification and sequencing of MHC class I sequences. (DOCX 16 kb) [file 12864_2016_2688_MOESM4_ESM.docx]

**Additional file 4 Table S2. Allele specific primers used for experimental MHC class I allele confirmation by Sanger sequencing**

| Name | Sequence (5'-3') |
| --- | --- |
| LfL#1017/F | CTCCTCCTGCTGCTCTCG |
| LfL#1018/F | TTTCACTTTCTCTCTCACAACCTG |
| LfL#1019/R | TCTAAAAATGTGAGGGGGAGAA |
| LfL#1020/R | CCACAGGGACCCCATTTT |
| LfL#1021/F | CCCAGGCTCCCACTCCC |
| LfL#1022/F | CCCCAGGCTCCCACTCT |
| LfL#1023/F | CCCAGGCTCCCACTCGA |
| LfL#1024/R | TACCTGCGCGCAGCAGCGT |
| LfL#1025/R | TACCTGCGCGCAGCAGCGC |
| LfL#1030/F | GCTCCCACTCCCTGAGC |
| LfL#1031/F | CCACTCCCTGAGGTATTTCC |
| LfL#1032/F | GAGCCCCGCTTCATCT |
| LfL#1033/R | AGCGTGTCCTTCCCGG |
| LfL#1034/R | CAGGTGTCTAAGGAGCCACTG |
| LfL#1035/R | GTGTCTGCGGAGCAACTT |
| LfL#1036/R | CCACTCCACGCACCG |
| LfL#1037/R | GCCCCTCCACGCACTC |
| LfL#1038/R | GCCACTCCACGCACGT |
| LfL#1039/R | GAGCCACTCCACGCACG |
| LfL#1040/F | TCCCACTCCCTGAGGTATTTCA |
| LfL#1041/R | GACCACCCGCGGTGATTAAAC |
| LfL#1042/F | CGGCTACTACAACCAGAGCGA |
