# Supplementary material for: A novel RNAseq–assisted method for MHC class I genotyping in a non-model species applied to a lethal vaccination-induced alloimmune disease
Source: BMC Genomics. 2016 May 17;17:365. doi: 10.1186/s12864-016-2688-0 (PMC4869273; doi:10.1186/s12864-016-2688-0)
Supplement: Additional file 7: Figure S4. — Classical MHC class I alleles: multiple alignment of amino acids. Alignment of the predicted amino acid sequences for classical MHC class I derived from the nucleotide sequence of alleles expressed in the data set. Dots: indicate identity to the first allele in the list; dashes: represent gaps compared to the first allele in the list; asterisk: denotes a stop codon; yellow labelled amino acids: uniquely occurring amino acid in an allele at a specific amino acid position in comparison to the other alleles. (DOCX 34 kb) [file 12864_2016_2688_MOESM7_ESM.docx]

Domain Leader Alpha 1

Amino acid 1 10 20 30 40 50 60

Allele

BoLA-1*03101 ---------FVLLLG--ALALTETRAGSHSLKYFYTAVSRPGDGEPRFITVGYVDDTQFVRFDSDAPDPRKEPRAPWIEKEGPEYWD

BoLA-1*06701_FBN_1 ---------......--..V...........R....G.....L.....FA........IA......RN..M......M.QK.....E

BoLA-2*00601_FBN_2 ---------IP..S.--V.V....V......R....G.....L......A.........T.......N..E......M.Q..L....

BoLA-2*00601_FBN_3 ---------IL..S.--V.V....V......R..S.......L......A.........T.......N..E......M.Q..L....

BoLA-2*01601 --------LLL..S.--V.V....V......R..S.......L......A.........T.......N..E......M.Q..L....

BoLA-2*01601_FBN_4 --------LLP..S.--V.V....V......R....G.....L......A.........T.......N..E......M.Q..L....

BoLA-2*01602_FBN_5 ---MRPRTLLL..S.--V.V....V......R..S.......L......A.........T.......N..E......M.Q..L....

BoLA-2*01802 ---------LL..SR--V.V....L......R..........L......A.........T.......N..D...V..M.Q.......

BoLA-2*02603 ---------LL..S.--V.V....L......R....G.....L......A.........T.......N..E...V..M.Q.......

BoLA-2*03201N ---------LL..S.--V.V....L.....*--------------------------------------------------------

BoLA-2*04501_FBN_6 ---MRPRTLLL..S.--V.V....L......R....G.....L......A....................T...VR...Q.......

BoLA-2*04801 ---MGPRTLLL..S.--V.V...........R..........L......S...............SAN..E......M.Q.......

BoLA-3*00401_FBN_7 ---MGPRALLL..S.--V.I...........R..S.......L....YLE..........Q......N..M....R.V.Q.......

BoLA-3*00402 ---MGPRALLL..S.--V.V...........R..S.......F....YLE..........Q......N..M....R.V.Q.......

BoLA-3*01001_FBN_8 ---MGPRTLLL..S.--V.V...........R..........L......S.....N.E............E...VR.M.Q..L....

BoLA-3*01101 ---MGPRTLLL..S.--V.V..........MR..S.......L....YLE.................N..M....R.V.Q.......

BoLA-3*01701 MRVMGPRTLLL..S.--V.V..........MR..S.......L....YLE................RN..M...KR.V.Q.......

BoLA-3*02702 ---MGPRTLLL..S.--V.V..........MR..S.......F....YLE................RN..M...TR.VKQ.......

BoLA-3*03301N ---------LL..S.--V.V...........R..S.......F....YLE..........Q......N..M....R.V.Q.......

BoLA-3*03301N_FBN_9 ---------LL..S.--V.V...........R..S.......F....YLE..........Q......N..M....R.V.Q.......

BoLA-3*05001 ---MGPRTLLL..S.--V.V..........MR..S.......L....YLE.................N..M...TR.VKQ.......

BoLA-4*06301_FBN_10 ---------LL..S.--V.V......................L......S.....N.E............T...VR.M.Q.......

BoLA-6*01402 ---------.M....--..V.I.......F.R..H.......LR..L...................R.......Q..M.........

BoLA-6*01501 --------L.M....--..V.I.........R....G.....LR..L...................R.......Q..M.........

BoLA-class_FBN_11 --------LLL..S.--V.V...........R.........DL....YLQ.................N..M....R.V.Q.......

BoLA-class_FBN_12 -------TLLL..S.--V.V...........R..H.......L......S...............SAN..E......M.Q.......

Alpha 2

70 80 90 100 110 120 130 140

BoLA-1*03101 DETRISKENTLLYRKNLNTLRGYYNQSEAGSHNIQAMFGCDVGPDGRFLRGYRQDAYDGRDYIALNEDLRSWTAADTAAQITKRKWE

BoLA-1*06701_FBN_1 EM..DA.KDQQRSQFC................TF.QI..........L.S..D.YG...............................

BoLA-2*00601_FBN_2 RN...Y.DTAQTF.VY................TL.W.S......G..L...FM.FG..........Q....................

BoLA-2*00601_FBN_3 RN...Y.DTAQTF.VY................TL.W.S.........L...FM.FG..........Q....................

BoLA-2*01601 RN...Y.DTAQTF.VY................TL.W.S.........L...FM.FG..........Q....................

BoLA-2*01601_FBN_4 RN...Y.DTAQTF.VY................TL.W.S......G..L...FM.FG..........Q....................

BoLA-2*01602_FBN_5 RN...Y.DTAQTF.VY................TL.W.S.........L...FM.FG..........Q....................

BoLA-2*01802 RN...Y.DTAQIF.A....AL...........TF.E.Y..Y......L.L.FM.F................................

BoLA-2*02603 R.......TAQTF.VD................T..E.Y..............E.YG.E.............................

BoLA-2*03201N ---------------------------------------------------------------------------------------

BoLA-2*04501_FBN_6 R...NL.DTAQTF.VD................T..E.Y........................L........................

BoLA-2*04801 EQ...V.DTAQSF.VG................TL.L.Y.............FM................................R.

BoLA-3*00401_FBN_7 RN..NA.G.AQSF.V.................TL.W.S.........LR..FM.YG......L...........GE.E.........

BoLA-3*00402 RN..NA.G.AQSF.V.................TL.W.S.........LR..FM.YG......L...........GE.E.........

BoLA-3*01001_FBN_8 RN...Y.DTAQTF.VD................TL.E.Y.........L.G..E.YG.E....L................H.S...V.

BoLA-3*01101 Q...KA.GTAQTF.A...IAL...........TF.W.Y.........LR..FM.YG...............................

BoLA-3*01701 R..QRA.G.AQIF.VS..N.............TF.W.Y.........L.G..E.YG..................GE.E.........

BoLA-3*02702 RN..NA.G.AQSF.VG................TL.W.S..Y......L...FM.YG......L..............E.........

BoLA-3*03301N RN..NA.G.AQFPSEPEHPA.--LL.P.R.RVSHPPVDVRLLRGA..ASPPRVHAVRLR.QRLPRP.RGPALLDRGGDGGS-DHQAQ

BoLA-3*03301N_FBN_9 RN..NA.G.VQFPSEPEHPA.--LL.P.R.RVSHPPVDVRLLRGA..ASPPRVHAVRLR.QRLPRP.RGPALLDRGGDGGS-DHQAQ

BoLA-3*05001 RN..NA.G.AQTF.GS..N.............TF.L.Y.........LR..FT.YG...........E...................

BoLA-4*06301_FBN_10 R...NF..TAQTF.V....................L.Y.........L...FT.FG......L........................

BoLA-6*01402 R..Q.....A.K..EA..I.............TY.R.Y.........L.S.FT.FG...............................

BoLA-6*01501 R..Q.....A.W..EA..N.............TL.L.Y.........L...FT.FG......L..................S...M.

BoLA-class_FBN_11 Q...KA.DAAQSS.VG..S.............TL.L.Y..Y.....SLR..FM.FG......L...........VE.V...S...M.

BoLA-class_FBN_12 EQ...V.DTAQSF.VD................TL.W.Y.............FM.........L........................

Alpha 3

150 160 170 180 190 200 210 220 230

BoLA-1*03101 AADYAESLRNYLEGTCVEWLRRYLENGKDTLLRADPPKAHVTHHPSSEREVTLRCWALGFYPEEISLTWQRSGEDQTQDMELVETRP

BoLA-1*06701_FBN_1 ..GE..RQ......E..............A...............I.D..............K........N...L...........

BoLA-2*00601_FBN_2 ...N...E......E..............................I.D.......................N...............

BoLA-2*00601_FBN_3 ...N...E......E..............A...............I.D.......................N...............

BoLA-2*01601 ...N...E......E...G..........A...............I.D.......................N...............

BoLA-2*01601_FBN_4 ...N...E......E...G..........A...............I.D.......................E...............

BoLA-2*01602_FBN_5 ...N...E......E..............A...............I.D.......................N...............

BoLA-2*01802 ..GE..RQ......R...G..........................I.D......................HE...............

BoLA-2*02603 ..............R...G..........A............R..I.........................E...............

BoLA-2*03201N ---------------------------------------------------------------------------------------

BoLA-2*04501_FBN_6 ..GE..RF...V..L...G..........................I.H.......................E...............

BoLA-2*04801 ..GA..RQ......R...G..........A...............I.........................E...............

BoLA-3*00401_FBN_7 ..G...VQ......E..............................I.G......................HD...............

BoLA-3*00402 ..G...VQ......E..........................A...I.D......................HD...............

BoLA-3*01001_FBN_8 ..GE..RF......R...G..........A...........A...I.D......................HD...............

BoLA-3*01101 ..GE..RQ.................T..................SI.GH..............D.......N...............

BoLA-3*01701 ..GA..RQ......R..............................I.........................N...............

BoLA-3*02702 .EG...VQ......E.......H......................I.D......................HN...............

BoLA-3*03301N VGGGRLR*-------------------------------------------------------------------------------

BoLA-3*03301N_FBN_9 VGGGRLR*-------------------------------------------------------------------------------

BoLA-3*05001 ..GE...W..............H......................I.DY......................N....M..........

BoLA-4*06301_FBN_10 ..GA...F...V..R...G..........................I.G...............D.......E...............

BoLA-6*01402 ..GE..RF...V..R..............A...............I..H......................N...............

BoLA-6*01501 ..GD..RQ......R...G.......................R.....H......................N...............

BoLA-class_FBN_11 ..GV..EQ......R..............................I.H.......................N...............

BoLA-class_FBN_12 ..GE..RD......R..............A...............I.........................E...............

240 250 260 270

BoLA-1*03101 SGDGNFQKWAALVVPSGEEQRYTCHVQHEGLQEPLTLRW

BoLA-1*06701_FBN_1 ........................R..............

BoLA-2*00601_FBN_2 ....T..........P........R..............

BoLA-2*00601_FBN_3 ....T...................R..............

BoLA-2*01601 ....T...................R..............

BoLA-2*01601_FBN_4 ....T.................M.R..............

BoLA-2*01602_FBN_5 ....T...................R..............

BoLA-2*01802 ....T...................R..............

BoLA-2*02603 ....T...................R......R.......

BoLA-2*03201N ---------------------------------------

BoLA-2*04501_FBN_6 ....T.......G...........R..............

BoLA-2*04801 ....T...................R..............

BoLA-3*00401_FBN_7 ....T............D......R..............

BoLA-3*00402 ....T............D......R..............

BoLA-3*01001_FBN_8 ....T............D......R..............

BoLA-3*01101 ....................K...R............K.

BoLA-3*01701 ....................K...R............K.

BoLA-3*02702 ....T...............K...R............K.

BoLA-3*03301N ---------------------------------------

BoLA-3*03301N_FBN_9 ---------------------------------------

BoLA-3*05001 ....................K...R............K.

BoLA-4*06301_FBN_10 ....T.......L.........R.R......P.......

BoLA-6*01402 ........................R..............

BoLA-6*01501 ........................R..............

BoLA-class_FBN_11 ....................K...R..............

BoLA-class_FBN_12 ....T...................R..............

Transmembrane domain Cytoplasmic domain

280 290 300 310 320 330

BoLA-1*03101 EPPQTSFLTMGIIVGLVLLVVA--VVAGAVIWRKKRSGEKRQTYTQAASGDSDQGSDVSLTVPKV*

BoLA-1*06701_FBN_1 ....P.......T.........--.........................................*

BoLA-2*00601_FBN_2 ........I.............--L...............GRI......S..T...........AV

BoLA-2*00601_FBN_3 ........I.............--L...............GRI......S..T...........AV

BoLA-2*01601 ......................--................GRI......S..A............*

BoLA-2*01601_FBN_4 ......................--................GRI......S..A............*

BoLA-2*01602_FBN_5 ........I.............--L...............GRI......S..T............*

BoLA-2*01802 ......................--................GRI......S..A............*

BoLA-2*02603 ......................--................GRI......S..A............*

BoLA-2*03201N ------------------------------------------------------------------

BoLA-2*04501_FBN_6 ......................--................GRI......N..A............*

BoLA-2*04801 ........I.............--L...............GRI......S..A............*

BoLA-3*00401_FBN_7 ....P................TGA....V..CM.......GGN.I..S.S..A............*

BoLA-3*00402 ....P................TGA....V..CM.......GGN.I..SGS..A............*

BoLA-3*01001_FBN_8 ....P................TGA....V..CM.......GGN.I..SGS..A............*

BoLA-3*01101 ....P................TGA....V..CM........G..I..S.S..A............*

BoLA-3*01701 ....P................TGA....V..CM........G..I..S.S..A............*

BoLA-3*02702 ....P................TGA....V..CM..P.....G..I..L.S..A............*

BoLA-3*03301N ------------------------------------------------------------------

BoLA-3*03301N_FBN_9 ------------------------------------------------------------------

BoLA-3*05001 ....P................TGA....V..CM........G..I..S.S..A............*

BoLA-4*06301_FBN_10 .....................TGA...........H....GGN.I..SGS..A............*

BoLA-6*01402 ......................--........M..H.....R.......N..A............*

BoLA-6*01501 ......................--........M..H.....R.......N..A............*

BoLA-class_FBN_11 ....P........I.......TGA....V..CM........G..I..S.S..A............*

BoLA-class_FBN_12 ......VPI....LV.......--L...............GRI......S..A............*
