# Supplementary material for: A novel RNAseq–assisted method for MHC class I genotyping in a non-model species applied to a lethal vaccination-induced alloimmune disease
Source: BMC Genomics. 2016 May 17;17:365. doi: 10.1186/s12864-016-2688-0 (PMC4869273; doi:10.1186/s12864-016-2688-0)
Supplement: Additional file 8: Figure S5. — Non-classical MHC class I alleles: multiple alignment of amino acids. Alignment of the predicted amino acid sequences for classical MHC class I derived from the nucleotide sequence of alleles expressed in the data set. Dots: indicate identity to the first allele in the list; dashes: represent gaps compared to the first allele in the list; asterisk: denotes a stop codon. (DOCX 33 kb) [file 12864_2016_2688_MOESM8_ESM.docx]

Domain Leader Alpha 1

Amino acid 10 20 30 40 50 60

Non-classical allele

BoLA-NC1*00101_FBN_13 --------LLLLLLGVLVPRDTRAGPHSMRYFLTAVSRPGLGEPRFITVGYVDDTQFVRFDSDRPDPRMEPRARWVEDEGPEYWD

BoLA-NC1*00201 ---------............................................................................

BoLA-NC1*00301_FBN_14 ---------................S..L..................I.....................................

BoLA-NC1*00401_FBN_15 ---------................S..L........................................................

BoLA-NC1*00601 ---------................S..L..................I.....................................

BoLA-NC1*00601_FBN_16 ---------................S..L..................I.....................................

BoLA-NC1*00601_FBN_17 ---------................S..L..................I.....................................

BoLA-NC1*00701_FBN_18 ---------................S..L..................A.....................................

BoLA-NC2*00101 MRVVGPRT.....P.A.ILTE....S..L...Y..............S...............A....I..T.....Q......H

BoLA-NC2*00101_FBN_19 MRVVGPRT.....P.A.ILTE....S..L...Y..............S...............A....I..T.....Q......H

BoLA-NC2*00101_FBN_20 MRVVGPRT.....P.A.ILTE....S..L...Y..............S...............A....I..T.....Q......H

BoLA-NC2*00102 ---------....P.A.ILTE.W..S..L...Y..............S...............A....I..T.....Q......H

BoLA-NC2*00102_FBN_21 ---------.....DA.ILTE....S..L...Y..............S...............A....I..T.....Q......H

BoLA-NC2*00102_FBN_22 ---------.....DA.ILTE....S..L...Y..............S...............A....I..T.....Q......H

BoLA-NC2*00102_FBN_23 ---------....P.A.ILTE.W..S..L...Y..............S...............A....I..T.....Q......H

BoLA-NC2*00103_FBN_24 ---------.....DA.ILTE....F..LK..Y..............S...............A....I..T.....Q......H

BoLA-NC3*00101 ---------....S....LTE.W..S..LS..N.G.YQ........FA.........A.....A.N.......P.M.Q......E

BoLA-NC3*00101_FBN_25 ---------....S....LTE.W..S..LS..N.G.YQ........FA.........A.....A.N.......P.M.Q......E

BoLA-NC3*00101_FBN_26 ---------....P....LTE.W..S..LS..N.G.YQ........FA.........A.....A.N.......P.M.Q......E

BoLA-NC4*00101 ---------....SE...LTE.W..S..LSC.C.C...........FA.........A.....A.N.......P.M.Q......E

BoLA-NC4*00101_FBN_27 ---------....S....LTE.W..S..LS..C.C...........FA.........A.....A.N.......P.M.Q......E

BoLA-NC4*00201 ---------....SE...LTE.W..S..LS..C.C...........FA.........A.....A.N.......P.M.Q......E

BoLA-NC4*00202_FBN_28 ---------....SE...LTE.W..Y..LS..C.C...........FA.........A.....A.N.......P.M.Q......E

BoLA-NC4*00202_FBN_29 ---------....SE...LTE.W..Y..LS..C.C...........FA.........A.....A.N.......P.M.Q......E

BoLA-NC4*00301_FBN_30 ---------....SE...LTE.W..S..LS..C.C...........FA.........A.....A.N.......P.M.Q......E

BoLA-NC5*00101_FBN_31 ---------...FSE...LTE.WT.Y..LN..C.CM.W...R.S..F.......M.IA.....Y.N.......P.M.Q.E....E

Alpha 2

70 80 90 100 110 120 130 140

BoLA-NC1*00101_FBN_13 QETRIQKENTQTFRANLNTLLGYYNQSEAGSHTIQWMHGCGVGSDGRLLRGYNQHAYDGKDYLALNEDLRSWTAADTAAQITKRK

BoLA-NC1*00201 .....................................................................................

BoLA-NC1*00301_FBN_14 .....................................................................................

BoLA-NC1*00401_FBN_15 .....................................................................................

BoLA-NC1*00601 ......................................................L...C..........................

BoLA-NC1*00601_FBN_16 ......................................................L...C..........................

BoLA-NC1*00601_FBN_17 ......................................................L...C..........................

BoLA-NC1*00701_FBN_18 ......................................................L........................H.S...

BoLA-NC2*00101 ...QRT.DTA.F..VY....R............V.E.Y..D..P..-.....D.F....R..I.................V..HN

BoLA-NC2*00101_FBN_19 ...QRT.DTA.F..VY....R............V.E.Y..D..P..-.....D.F....R..I.................V..HN

BoLA-NC2*00101_FBN_20 ...QRT.DTA.F..VY....R............V.E.Y..D..P..-.....D.F....R..I.................V..HN

BoLA-NC2*00102 ...QRT.DTA.F..VY....R............V.E.Y..D..P..-.....D.F....R..I.................V..HN

BoLA-NC2*00102_FBN_21 ...QRT.DTA.F..VY....R............V.E.Y..D..P..-.....D.F....R..I.................V..HN

BoLA-NC2*00102_FBN_22 ...QRT.DTA.F..VY....R............V.E.Y..D..P..-.....D.F....R..I.................V..HN

BoLA-NC2*00102_FBN_23 ...QRT.DTA.F..VY....R............V.E.Y..D..P..-.....D.F....R..I.................V..HN

BoLA-NC2*00103_FBN_24 ...QRT.DTA.F..VY....R............V.E.Y..D..P..-.....D.F....R..I.................V..HN

BoLA-NC3*00101 EM..DA..SQ.ES.LC.YN.R........E..IL.V.F..E..P.......FW.K....R..I..............V.......

BoLA-NC3*00101_FBN_25 EM..DA..SQ.KS.LC.YN.R........E..IL.V.F..E..P.......FW.K....R..I..............V.......

BoLA-NC3*00101_FBN_26 EM..DA..SQ.ES.LC.YN.R........E..IL.V.F..E..P.......FW.K....R..I..............V.......

BoLA-NC4*00101 AM..DA.KAQ.RL.TG...IR.F..........L..VL..D..PE......IW.N....A...............N.V.......

BoLA-NC4*00101_FBN_27 AM..DA.KAQ.RL.TG...IR.F..........L..VL..D..PE......IW.N....A...............N.V.......

BoLA-NC4*00201 AM..DA.KAQ.RL.TG...IR.F..........L..VL..D..L.......IW.N....A...............N.V.......

BoLA-NC4*00202_FBN_28 AM..DA.KAQ.RL.TG...IR.F..........L..VL..D..PE......IW.N....A...............N.V.......

BoLA-NC4*00202_FBN_29 AM..DA.KAQ.RL.TG....R.F..........L..VF..D..PE......IW.N....A...............N.V.......

BoLA-NC4*00301_FBN_30 AM..DA.KAQ.RL.TG....R.F..........L..VF..D..PE......IW.N....A...............N.V.......

BoLA-NC5*00101_FBN_31 EV.GSA..AQRRM.LE...MR.F.......F..L.FVF..D..PE......FW.K....AY.IS...........N........R

Alpha 3

150 160 170 180 190 200 210 220 230

BoLA-NC1*00101_FBN_13 WEATGAAERFRNYLEGKCVKLLRRHLENGKDTLLRADPPMAHVTHHPISEREVTLRCWALGFYPEEISLTWQRNGEDQTQDMELV

BoLA-NC1*00201 ..................................................H..................................

BoLA-NC1*00301_FBN_14 .....................................................................................

BoLA-NC1*00401_FBN_15 ...A.E..........E......................K...A...S.....................................

BoLA-NC1*00601 ...A.E..........E..EW.......R..A.......K.........D......................HD...........

BoLA-NC1*00601_FBN_16 ...A.E..........E..EW.......R..A.......K.........D......................HD...........

BoLA-NC1*00601_FBN_17 ...A.E..........E..EW.......R..A.......K...A.....D......................HD...........

BoLA-NC1*00701_FBN_18 ...............................A.......K......S..G...................................

BoLA-NC2*00101 A..A.D.A.V.I.......EW...Y.VT...........KT..A.....DH.............D........D...........

BoLA-NC2*00101_FBN_19 A..A.D.A.V.I.......EW...Y.VT...........KT..A.....DH.............D........D...........

BoLA-NC2*00101_FBN_20 A..A.D.A.V.I.......EW...Y.VT...........KT..A..R..D..............D........D...........

BoLA-NC2*00102 A..A.D.A.V.I.......EW...Y.VT...........KT..A.....DH.............D........D...........

BoLA-NC2*00102_FBN_21 A..A.D.A.V.I.......EW...Y.VT...........KT..A..R..D..............N........D...........

BoLA-NC2*00102_FBN_22 A..A.D.A.V.I.......EW...Y.VT...........KT..A.....D.......................D...........

BoLA-NC2*00102_FBN_23 A..A.D.A.V.I.......EW...Y.VT...........KT..A..R..D..............D........D...........

BoLA-NC2*00103_FBN_24 A..A.D.A.V.I.......EW...Y.VT...........KT..A.....DH.............D........D...........

BoLA-NC3*00101 .DVS.Q.KIQ.....V...QW.L....T...........KT..A..R..D..............K..T....QD...L.......

BoLA-NC3*00101_FBN_25 .DVS.Q.KIQ.....V...QW.L....T...........KT..A.....D..............K..T....QD...L.......

BoLA-NC3*00101_FBN_26 .DVS.Q.KIQ.....V...QW.L....T...........KT..A..R..D..............K..T....QD...L.......

BoLA-NC4*00101 ..TS.E..FQ.....V...QW.L....T...........KT..A.....D.......................D...........

BoLA-NC4*00101_FBN_27 ..TS.E..FQ.....V...QW.L....T...........KT..A.....D.......................D...........

BoLA-NC4*00201 ..TS.E..FQ.....V...QW.L....T...........KT..A.....D......................CD...........

BoLA-NC4*00202_FBN_28 ..TS.E..FQ.....V...QW.L....T...........K...A.....D.......................D...........

BoLA-NC4*00202_FBN_29 ..TS.E..FQ.....V...QW.L....T...........K...A.....D.......................D...........

BoLA-NC4*00301_FBN_30 ..TS.E..FQ.....V...QW.L....T...........KT..A.....D.......................D...........

BoLA-NC5*00101_FBN_31 ..IS.E.DFQ.....VI..QW.LS...KW..........KT.....R..D..............K..T.....D...........

240 250 260 270

BoLA-NC1*00101_FBN_13 ETRPSGDGTFQKWAALAVPSGEEQRYTCRVQHEGLQEPLTLRW

BoLA-NC1*00201 .........................YTCRVQHEGLQEPLTLRW

BoLA-NC1*00301_FBN_14 .........................YTCRVQHEGLQEPLTLRW

BoLA-NC1*00401_FBN_15 ....................................G......

BoLA-NC1*00601 .........................YTCRVQHEGLQEPLTLRW

BoLA-NC1*00601_FBN_16 .........................YTCRVQHEGLQEPLTLRW

BoLA-NC1*00601_FBN_17 .........................YTCRVQHEGLQEPLTLRW

BoLA-NC1*00701_FBN_18 .........................YTCRVQHEGLQEPLTLRW

BoLA-NC2*00101 ................V...........H..............

BoLA-NC2*00101_FBN_19 ................V...........H..............

BoLA-NC2*00101_FBN_20 ................V...........H..............

BoLA-NC2*00102 ................V...........H..............

BoLA-NC2*00102_FBN_21 ................V...........Y..............

BoLA-NC2*00102_FBN_22 ................V...........H..............

BoLA-NC2*00102_FBN_23 ................V...........H..............

BoLA-NC2*00103_FBN_24 ................V...........H..............

BoLA-NC3*00101 ................V..........................

BoLA-NC3*00101_FBN_25 ................V..........................

BoLA-NC3*00101_FBN_26 ................V..........................

BoLA-NC4*00101 ................V...........H..........S...

BoLA-NC4*00101_FBN_27 ................V...........H..........S...

BoLA-NC4*00201 ................V...........H..........S...

BoLA-NC4*00202_FBN_28 ................V...........H..........S...

BoLA-NC4*00202_FBN_29 ................V...........H..........S...

BoLA-NC4*00301_FBN_30 ................V...........H..........S...

BoLA-NC5*00101_FBN_31 .............V..V...........H..........S...

Transmembrane domain Cytoplasmic domain

280 290 300 310 320 330 340

BoLA-NC1*00101_FBN_13 EPPQPSVPIIGIILVLVLL-VV--AVVAGAVIWSKKRSGEKGRIYT*ASSSDSAQGSDVSLTVPKES

BoLA-NC1*00201 ----------.........-..--......................*..................V*

BoLA-NC1*00301_FBN_14 .........M.........-..--.............*........Q..................V*

BoLA-NC1*00401_FBN_15 --------------------------------------........Q....................

BoLA-NC1*00601 .........M.....P...-..--.............*........Q..............M...V*

BoLA-NC1*00601_FBN_16 .........M.........-..--.............*........Q..............M.....

BoLA-NC1*00601_FBN_17 .........M.........-..--.............*........Q..............M...V*

BoLA-NC1*00701_FBN_18 .........M.........-..--........*.............Q....................

BoLA-NC2*00101 ......I..M...VG....-M.TG...T..M..R..H......G..QTA..Y.D.......M...V*

BoLA-NC2*00101_FBN_19 ......I..M...VG....-M.TG...T..M..R..H...R..G..QTA..Y.D.......M...V*

BoLA-NC2*00101_FBN_20 ......I..M...VG....-M.TG...T..M..R..H......G..QTA..Y.D.......M...V*

BoLA-NC2*00102 ......I..M...VG....-M.TG...T..M..R..H......G..QTA..Y.D.......M...V*

BoLA-NC2*00102_FBN_21 ......IL.M...VG....-M.TG...T.....R..H......G..QTA..Y.D.......M...V*

BoLA-NC2*00102_FBN_22 ......I..M...VG..H.-M.TG.M.T.....R..H........SQ......D.....P.....V*

BoLA-NC2*00102_FBN_23 ......I..M...VG....-M.TG...T..M..R..H......G..QTA..Y.D.......M...V*

BoLA-NC2*00103_FBN_24 .....CI..M...VG....-M.TG...T.....R..H......G..QTA..Y.D...........V*

BoLA-NC3*00101 ....L..L.K...VG.I..-M.TG...T.....R..H......G..QTA.....*..........G*

BoLA-NC3*00101_FBN_25 ....L..L.K...VG.I..-M.TG...T.....R..H......G..QTA....D...........V*

BoLA-NC3*00101_FBN_26 ....L..L.K...VG.I..-M.TG...T.....R..H......G..QTA.....*..........G*

BoLA-NC4*00101 K.....I..MD..VG....-M.TG...T.....R..H..QI.KG..QSA............M...V*

BoLA-NC4*00101_FBN_27 K.....I..MD..VG....-M.TG...T.....R..H..QI.KG..QSA............M...V*

BoLA-NC4*00201 K.....I..M...VG....-M.TG...T.....R..H..QT..G..Q.A............M...V*

BoLA-NC4*00202_FBN_28 K.....I..M...VG....-M.TG...T.....R..HA.QT..G..Q.A............M...V*

BoLA-NC4*00202_FBN_29 K.....I..M...VG....-M.TG...T.....R..H..QT..G..Q.A...R........M...V*

BoLA-NC4*00301_FBN_30 K.....I..M...VG....-M.TG...T.....R..H..QT..G..Q.A...R........M...V*

BoLA-NC5*00101_FBN_31 ......I......VG....LM.TG...T.....R..H...T..G..Q.EI...D.......A...V*
